# The usefulness of a checklist approach-based confirmation scheme in identifying unreliable COVID-19-related health information: a case study in Japan

**DOI:** 10.1057/s41599-022-01293-3

**Published:** 2022-08-15

**Authors:** Nanae Tanemura, Tsuyoshi Chiba

**Affiliations:** National Institutes of Biomedical Innovation, Health and Nutrition, Tokyo, Japan

**Keywords:** Education, Social policy

## Abstract

Consumers are increasingly able to easily access health information online about food products. However, consumers have difficulty identifying reliable health information from diverse sources along with information about the coronavirus disease (COVID-19) pandemic because the inundation of information (both true and false) overwhelm consumers. We investigated the usefulness of a checklist confirmation scheme for identifying unreliable COVID-19-related health information. Data were collected from June 30–July 1, 2021. First, we measured 700 participants’ baseline health literacy levels by having them read unreliable health information about the efficacy of green tea intake in preventing COVID-19 based on the results of animal experimentation. Second, participants read an explanation with a five-step flowchart of how to identify reliable health information. Thereafter, we remeasured participants’ health literacy levels. To identify the factors hindering the effect of the confirmation scheme, a logistic regression analysis was performed to calculate odds ratios (ORs) and 95% confidence intervals (CIs). Overall, 77.9% (293/376) of those with low health literacy levels at baseline still had low literacy after the intervention. The factor that hindered the confirmation scheme’s usefulness was benefit perceptions of food ingredients (OR: 0.493; 95% CI: 0.252–0.966). Consumers with higher benefit perceptions of a target product faced more difficulties using the confirmation scheme effectively. Therefore, the most effective strategies involve filtering information at the organizational level rather than the individual level, which should help consumers correctly identify misinformation concerning food and health and promote accurate decision-making.

## Introduction

In recent years, consumers have been able to easily access diverse sources of health information via mass media owing to increased Internet use. Acquiring knowledge from highly accessible mass media and the Internet is easy. Though health information is abundant, the credibility of such information is uncertain (Zhang et al., 2015). This issue has been exacerbated by the spread of coronavirus disease 2019 (COVID-19), and has accordingly been labeled by the World Health Organization (WHO, 2020) as an “infodemic”—a combination of the terms “information” and “epidemic.” The infodemic is represented by the greater difficulty that consumers experience in finding and selecting reliable information sources regarding the best ways to cope with health problems (Luk et al., 2021). The United Nations emphasized the need to develop an emergency strategy to cope with this infodemic, considering the global spread of COVID-19 (WHO, 2020). Furthermore, in Japan, the Consumer Affairs Agency, Government of Japan (2021), issued a request to modify health food labels that claim to have preventive effects against COVID-19 in response to the spread of the infection (Consumer Affairs Agency, 2021). Moreover, the agency alerted consumers about unreliable information related to healthy foods being shared through social networking services in 2021 (Consumer Affairs Agency, 2021).

One way to curtail the spread of misinformation is to enable users to vet health information they can trust. Therefore, confirmation tools need to be developed at the individual level because automatic detection methods cannot completely filter health-related misinformation (Cuan-Baltazar et al., 2020). The rapidly growing need for consumers to select reliable health information from ample information with careful critical consideration is an important focus area and one way to limit the spread of misinformation (Li et al., 2022). Confirmation tools are necessary to utilize information from the mass media to acquire the latest knowledge in keeping with scientific and technological advancements and changes in the social environment, as knowledge from compulsory education has now become outdated (Kusumi, 2013). Thus, skills for selecting appropriate information via the Internet when seeking new knowledge in response to scientific and technological advances are required (Li et al., 2022). Based on the definition provided by the National Institute of Health (NIH), these skills, termed health literacy, are described by the Japanese Health Literacy Association as follows: “The ability to find, understand, and use information and services to inform health-related decisions and actions for themselves and others” (NIH, 2021).

The characteristics of information differ between health misinformation and false news. As a result, new tools are needed to help users identify health misinformation. Although the “checklist approach” to answer a list of questions for each criterion is a popular evaluation method for identifying misinformation, it may not be practical or effective (Meola, 2004). If this checklist approach is used, the checklist should be shortened (Metzger, 2007). Considering the importance of literacy in determining reliable health information, Yoshitaka Tsubono proposed a flowchart with steps that can help consumers evaluate the reliability of health information (please see Supplementary Information: “Explanation with a five-step flowchart to identify reliable health information”) (Tsubono, 2002). With the implementation of each step in the checklist, information becomes increasingly reliable. The revised five-step flowchart was made accessible in public spaces in a comprehensive manner for effective use among consumers (National Institutes of Biomedical Innovation, 2021b). However, as knowledge-based supplementation alone is insufficient (Kikkawa and Kinoshita, 1989), it is important to identify the factors that hinder the confirmation scheme’s usefulness among consumers.

Therefore, we examine the usefulness of the developed confirmation scheme for identifying unreliable COVID-19-related health information. We also ascertain the factors that hinder the confirmation scheme’s usefulness in identifying unreliable health information.

## Methods

### Study sample and selection process

An online survey was administered to 700 participants from June 30 to July 1, 2021. It was conducted by Cross Marketing Inc (Tokyo, Japan) in Japan. We recruited participants from survey panels representative of registered consumers in Cross Marketing Inc. The inclusion criteria were participants aged ≥20 years with no education or professional work experience related to medicine or nutrition after high-school graduation (e.g., working as a chef, registered dietitian, adviser, food developer, etc.). The participants confirmed their eligibility by answering questions on a screening page before taking the survey. Finally, we enrolled the eligible participants by sex and age according to Japan’s population distribution (Ministry of Internal Affairs and Communications, 2016). No participant was excluded.

### Questionnaire

The questionnaire comprised four major domains: (A) whether green tea intake can prevent COVID-19 after reading the test health information (one question); (B) the selected step out of the five steps (one question); (C) whether green tea intake can prevent COVID-19 after the explanation with the five-step flowchart for identifying reliable health information (one question); and (D) participant demographics such as age, sex, education level, frequency of green tea intake, benefit perceptions, and numeracy score (i.e., ability to understand numeric information; seven questions) according to the survey flow (see Supplementary Information). Data were collected using Likert-type scale responses ranging from 1 (*low*) to 7 (*high*) for benefit perceptions, and the numeracy score ranging from 1 (*low*) to 6 (*high*) (see Supplementary Information). Both sets of responses were scored using descriptive statistics, and higher scores indicated a higher benefit perception and higher numeracy scores. Furthermore, both sets of scores were classified into two categories (low/high) using the median as the classification parameter.

### Test health information related to the efficacy of green tea in preventing COVID-19

The test health information provided to participants was related to the efficacy of green tea intake in preventing COVID-19. This information was presented as an article based on the results of animal experimentation; however, the article and details provided in it were on testing health information (Ministry of Agriculture Forestry and Fisheries of Japan, 2021b) (see Supplementary Information for further details). In 2019, green tea intake was ranked fourth among beverages consumed in Japan. Currently, the frequency of green tea intake has increased by 14.3% owing to its perceived efficacy in preventing COVID-19 (Ministry of Agriculture Forestry and Fisheries of Japan, 2021a). However, there were no scientific data to provide evidence that green tea intake prevents the spread of COVID-19 (National Institutes of Biomedical Innovation, 2021b). Therefore, this topic is appropriate as test health information to further explore and emphasize the issues relating to the COVID-19 infodemic.

### Explaining the five-step flowchart for identifying reliable health information

An explanation and a five-step flowchart as a confirmation scheme were used to identify reliable health information (see Supplementary Information for further details). If the health information met the criteria in all five steps, it was deemed to have sufficient scientific evidence to indicate a health effect. The test health information used in this survey only met the criteria outlined in the first step, thus showing a lack of scientific evidence.

### Definition

#### Health literacy level

The health information about green tea presented in this survey alone does not indicate that it is effective for people. Nonetheless, if a respondent answered “yes” to the question, “Do you think green tea has a health effect in preventing COVID-19?” incorrectly, we classified that respondent’s health literacy level as low. On the contrary, respondents who could identify the health effects on people were uncertain, that is, those who answered “no,” were classified as having a high health literacy level.

#### Selection skill

“Selection skill” in this survey was defined as the skill to identify reliable health information using a five-step flowchart without any expert explanations. Depending on the participants’ responses indicating selected/not selected in the first step in the flowchart in the (1) Main survey of the questionnaire, they were categorized as having a high/low selection skill regarding health information.

### Data collection

This survey was conducted with study panels via a website operated by Cross Marketing Inc, which is a contract research outsourcing company in Japan. A description of the study’s outline and objectives was provided on the webpage to inform participants. Completing the survey was regarded as providing voluntary consent based on the local and standard ethical guidelines published in Japan.

### Study outcomes

This cross-sectional study examined two outcomes: (1) the usefulness of the developed confirmation scheme for identifying unreliable COVID-19-related health information: the number of consumers with a low health literacy level after the explanation, and (2) the factors that hindered the confirmation scheme’s usefulness.

### Analysis

We analyzed 700 cases and summarized their demographic data. The differences between the categories were tested using Fisher’s exact tests for categorical variables. The number of consumers with low health literacy after the explanation was calculated. The factors that hindered the confirmation scheme’s usefulness were investigated using multivariate logistic regression analysis (simultaneous forced entry) to calculate odds ratios (ORs) and 95% confidence intervals (CIs). Demographic data, such as age, sex, education level, frequency of green tea intake, benefit perception of green tea, numeracy score, and health literacy level at baseline, were input as explanatory variables into this model, with a two-sided alpha level of *p* < 0.2, considering the lack of preliminary findings on the factor of this study outcome. The variance inflation factor (VIF) was used to check for multicollinearity, where VIF < 10 indicated no overall multicollinearity.

We performed all statistical analyses using the statistical application software EZR (Kanda, 2013), a modified version of the R Commander that adds statistical functions frequently used in biostatistics. *p* < 0.05 (two-sided) was deemed significant.

## Results

A total of 700 participants completed the survey; we did not exclude any participants from the analysis. The mean age of the participants was 45.8 years (standard deviation [SD] = 15.1) (86.7% [*n* = 607] were aged <65 years, and 13.3% [*n* = 93] were aged ≥65 years). The most frequently used sources of information were the TV (60.1%, *n* = 421) and the Internet (58.9%, *n* = 412). Regarding intake frequency, 31.1% (*n* = 218) drank green tea daily, and 47.6% (*n* = 333) drank it sometimes. The mean score of benefit perceptions was 4.89 (SD = 1.37; Table 1). The proportion of consumers with a low health literacy level was 53.7% (376 of 700) at baseline. There were significant differences in sex (*p* = 0.001), frequency of green tea intake (*p* < 0.001), benefit perceptions (*p* < 0.001), and numeracy score group (*p* < 0.001). The highest proportion of consumers with a low health literacy level was found in 61.9% (135 of 218) of those who drink green tea daily, but the lowest proportion, 29.6% (45 of 152), was found in the low benefit perception group (Table 2).

**Table 1:** Characteristics of the study sample (*N* = 700).

Characteristics	*n*	%
Age		
Mean (SD)	45.8	(15.1)
Under 65 years	607	86.7
65 years or older	93	13.3
Sex		
Male	350	50.0
Female	350	50.0
Education level		
Junior high/high-school graduate	214	30.6
Junior college graduate or higher	486	69.4
Source of information		
TV	421	60.1
Radio	61	8.7
Newspaper, magazine, advertisement	185	26.4
Internet	412	58.9
Social networking service	87	12.4
Store front	96	13.7
Hospital	126	18.0
Pharmacy	76	10.9
Drugstore	127	18.1
Contact the company	5	0.7
Family	161	23.0
Friends/acquaintances	125	17.9
Other	81	11.6
Frequency of green tea intake		
Daily	218	31.1
Sometimes	333	47.6
Do not drink	149	21.3
Benefit perceptions		
Mean (SD)	4.89	(1.37)
Median (min–max)	5.00	(1–7)
^a^ Low	152	21.7
High	548	78.3
Numeracy score		
Mean (SD)	4.30	(1.08)
Median (min–max)	4.25	(1–6)
^b^ Low	322	46.0
High	378	54.0

*SD* standard deviation.

^a^Low < 5.00 ≤ high.

^b^Low < 4.25 ≤ high.

**Table 2:** Status of consumers’ health literacy level at baseline (*N* = 700).

Characteristics	*N*	Low		High		*p*-value
		*n*	%	*n*	%	
All						
	700	376	53.7	324	46.3	
Age (years)						
Under 65	607	321	52.9	286	47.1	
65 or older	93	55	59.1	38	40.9	0.267
Sex						
Male	350	166	47.4	184	52.6	
Female	350	210	60.0	140	40.0	0.001
Education level						
Junior high/high-school graduate	214	117	54.7	97	45.3	
Junior college graduate or higher	486	259	53.3	227	46.7	0.743
Frequency of green tea intake						
Daily	218	135	61.9	83	38.1	
Sometimes	333	185	55.6	148	44.4	
Do not drink	149	56	37.6	93	62.4	<0.001
Benefit perceptions^a^						
Low	152	45	29.6	107	70.4	
High	548	331	60.4	217	39.6	<0.001
Numeracy score^b^						
Low	222	147	45.7	175	54.3	
High	378	229	60.6	149	39.4	<0.001
Selection skill						
Low	348	193	55.5	155	44.5	
High	352	183	52.0	169	48.0	0.364

^a^Low < 5.00 ≤ high.

^b^Low < 4.25 ≤ high.

### Usefulness of the developed confirmation scheme for identifying unreliable COVID-19-related health information

The proportion of consumers with a low health literacy level at baseline who also had the same literacy level after the explanation was 77.9% (293/376). Among the low health literacy group at baseline, there was a significant difference in benefit perceptions (*p* = 0.033; Table 3).

**Table 3:** Usefulness of the developed confirmation scheme for identifying unreliable COVID-19-related health information (*N* = 376)^a^.

Characteristics	Health literacy level (after explanation)				
	Low		High		*p*-value
	*n*	%	*n*	%	
All	293	77.9	83	22.1	
Age (years)					
Under 65	246	76.6	75	23.4	0.163
65 or older	47	85.5	8	14.5	
Sex					
Male	129	77.7	37	22.3	1
Female	164	78.1	46	21.9	
Education level					
Junior high/high-school graduate	94	80.3		19.7	0.503
Junior college graduate or higher	199	76.8		23.2	
Frequency of green tea intake					
Daily	110	81.5	25	18.5	0.457
Sometimes	140	75.7	45	24.3	
Do not drink	43	76.8	13	23.2	
Benefit perceptions^b^					
Low	29	64.4	16	35.6	0.033
High	264	79.8	16	35.6	
Numeracy score^c^					
Low	113	76.9	34	23.1	0.704
High	180	78.6	49	21.4	
Selection skill					
Low	157	81.3	36	18.7	0.107
High	136	74.3	47	25.7	

^a^Consumers with low literacy level at baseline.

^b^Low < 5.00 ≤ high.

^c^Low < 4.25 ≤ high.

### Factors that hindered the confirmation scheme’s usefulness

The VIF was employed to check for multicollinearity. None of the VIF values were up to 10, indicating no collinearity in the model. The VIF values for age, benefit perceptions, and selection skills were 1.00 in all variables. Table 4 displays the results of the logistic regression analysis to identify the factors that hindered the educational intervention. The only significant factor found was benefit perceptions (OR: 0.493; 95% CI: 0.252–0.966; Table 4).

**Table 4:** Factors that hinder the usefulness of the confirmation scheme (*N* = 376)^a^.

Variable	Adjusted OR	(95% CI)	*p*-value
Age (years)			
Under 65	Reference		
65 or older	0.607	(0.273–1.320)	0.221
Benefit perceptions^b^			
Low	Reference		
High	0.493	(0.252–0.966)	0.039
Selection skill			
Low	Reference		
High	1.440	(0.877–2.370)	0.150

*OR* odds ratio, *CI* confidence interval.

^a^Consumers with low literacy level at baseline.

^b^Low < 5.00 ≤ high.

## Discussion

### Demographics of the study population

The mean reported numeracy score was high; this may be linked to the study participants’ high education levels. Approximately 78.7% of participants consumed green tea sometimes or daily. Reportedly, 82% of Japanese people drank green tea almost daily in a 2005 study (Ministry of Agriculture Forestry and Fisheries of Japan, 2005); thus, the current sample can be considered representative of the Japanese population in adhering to traditional customs.

In previous reports, the TV and the Internet were the main information sources affecting eating habits (Ministry of Health Labour and Welfare, 2020; Keene Woods et al., 2021), and these results are supported in our study, where the TV and the Internet were the main sources of health information. Furthermore, another national survey by the Ministry of Agriculture, Forestry, and Fisheries of Japan reported that the primary source of information on green tea intake was the TV (Ministry of Agriculture Forestry and Fisheries of Japan, 2005).

A large amount of unreliable health information has emerged on the Internet, especially following the global spread of COVID-19, thus triggering an “Infodemic” (WHO, 2021). Therefore, it is important to identify reliable health information from the perspective of public health control measures.

### Usefulness of the developed confirmation scheme for identifying unreliable COVID-19-related health information

Most participants had a low health literacy level even after an explanation with a flowchart with five steps for identifying reliable health information about the efficacy of green tea intake in preventing COVID-19.

**Fig. 1:**
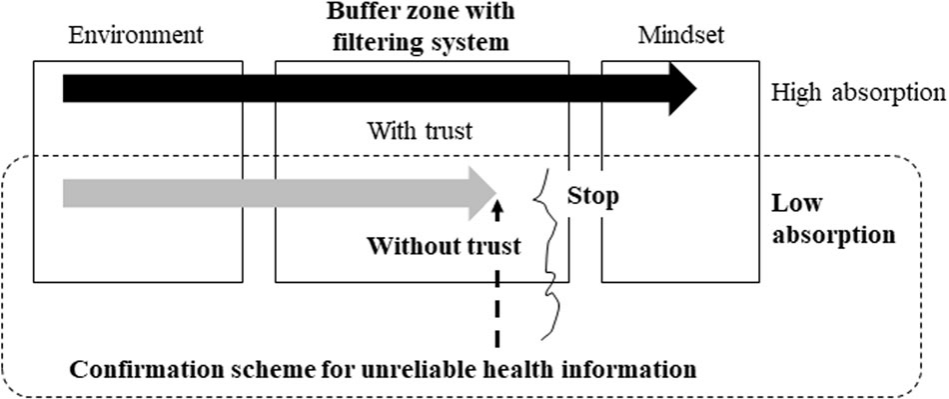
Information absorption process.

The “mindsponge mechanism” theory (Vuong and Napier, 2015; Vuong, 2022) states that the presence or absence of trust affects information absorption after moving to the filtering system stage (Fig. 1). While accessing online health information, people judge its value with a trust-based subjective evaluation (Wathen and Burkell, 2002; Vuong et al., 2022b). This determines the perceived benefit of the information and affects people’s behavior accordingly. If this developed confirmation scheme for identifying unreliable COVID-19-related health information made consumers aware that health information is unreliable, it may have suppressed the false perception that green tea is effective against the new coronavirus. However, the explanations with this confirmation scheme in this individual-level information filtering system proved to be insufficient for consumers.

A previous study on health literacy in Japan investigated the actual state of literacy among 134 nutrition teachers and dietitians (Gamo, 2011). In total, 76 of the 134 experts could not make a correct decision after reading the example of health information provided. However, 72 out of 76 participants changed their decisions correctly after using Yoshitaka Tsubono’s checklist (please see Supplementary Information for the flowchart). This flowchart contains several steps that are effective in allowing health professionals to objectively interpret health information; however, based on our survey results, it may not be useful for consumers except for those with high health literacy and education levels. Therefore, future studies should be conducted to develop suitable explanations for this confirmation scheme in consideration of consumers with low health literacy levels. For example, these interventions can include developing headings that are easier for consumers to understand (DeRosa et al., 2021), which may help them to follow the intervention better and identify reliable health information more effectively.

### Factors that hinder the confirmation scheme’s usefulness

This survey revealed that having a higher benefit perception was the main factor that hindered the confirmation scheme’s usefulness. Higher perceived benefits of the object of communication (e.g., green tea in this survey) can mitigate the usefulness of health promotion campaigns. This could be because diverse information sources comprise a mixture of reliable and unreliable health information and their high benefit perceptions could influence information vetting (Tangcharoensathien et al., 2020). The NIH also pointed out that “Everyone, no matter how educated, is at risk for misunderstanding health information if the topic is emotionally charged or complex” (NIH, 2021).

**Fig. 2:**
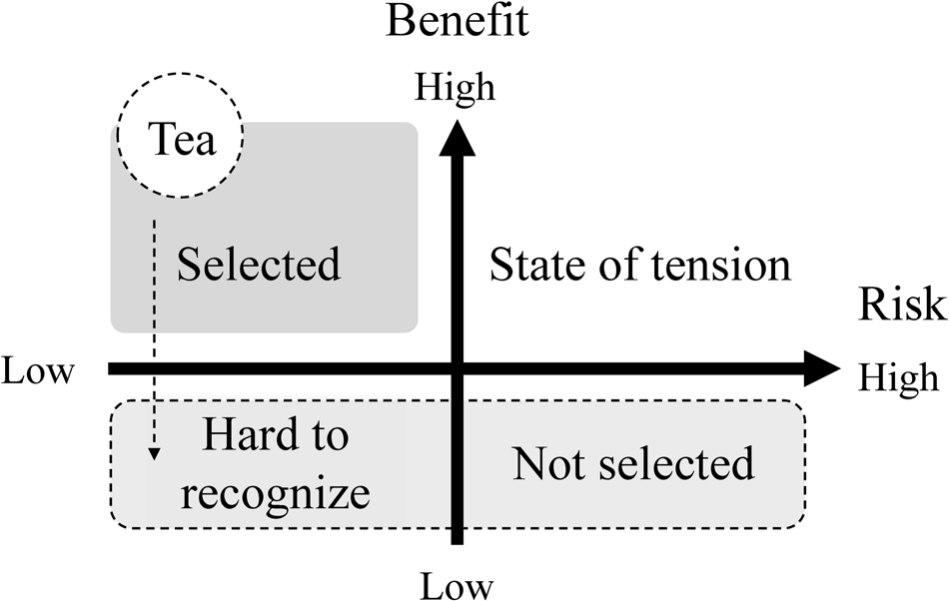
Informed decision-making in light of the perceived risks and benefits.

In Japan, most consumers drink green tea in their daily lives (Ministry of Agriculture Forestry and Fisheries of Japan, 2005); therefore, they might have a higher expectation of the health benefits of green tea intake at baseline. In general, the balance between benefits and risks is connected to consumers’ informed decision-making (Tsuchida and Itoh, 2003). Targeting products that garner high benefit perceptions and low-risk perceptions can help people with safe selection and decision-making (Fig. 2). The level of a consumer’s perception of risks and benefits is linked to their final informed decision-making skills. Therefore, an updated confirmation scheme paying attention to the consumer’s perception correctly are key points for promoting effective health communication with non-expert groups (Tsuchida and Itoh, 2003).

Therefore, the efforts of both producers and consumers of health information are needed to facilitate health promotion (He et al., 2021), which may enable people to improve their health considering the risk/benefit perceptions of non-experts. This suggests that the importance of “organizational health literacy”—the degree that organizations equitably enable individuals to find, understand, and use information and services to inform health-related decisions and actions for themselves and others (NIH, 2021)—as well as “personal health literacy” should receive more attention in the future.

During the COVID-19 pandemic, a great deal of misinformation has increased the burden of individual-level information filtering in the “mindsponge mechanism” theory. Therefore, information filtering at the organizational level is also an important aspect of the knowledge management framework for public health measures in a crisis (Zarocostas, 2020; Vuong et al., 2022a). Currently, the National Institutes of Biomedical Innovation provides health information to consumers after confirming with trusted information sources the existence of scientific evidence for the efficacy of healthy foods that are said to be “effective in preventing novel coronaviruses” (National Institutes of Biomedical Innovation, 2021a). Such efforts at the organizational level are important for public organizations to promote risk communication during emergencies (Cuan-Baltazar et al., 2020).

### Strengths and limitations

This was the first Japanese survey to examine the usefulness of the developed confirmation scheme for identifying unreliable COVID-19-related health information with the five-step flowchart proposed by Yoshitaka Tsubono. However, most participants were aged <65 years; hence, the results may not be generalizable to the greater Japanese population. Nevertheless, this study sample can be generalized with some caution because we recruited nationally representative consumers in terms of age, sex, and demographic composition.

## Conclusion

Explanations using the confirmation scheme, which used the “checklist approach” for identifying unreliable COVID-19-related health information on food, proved insufficient for consumers. However, benefit perceptions are crucial in facilitating appropriate decision-making, especially in low health literacy level groups. Therefore, future research should focus on the development of the most effective strategies in organizational-level information filtering while considering consumers’ benefit perceptions to help them correctly identify misinformation regarding food and health.

## Supplementary information

Supplementary Information

## Data Availability

The authors confirm that the data supporting the findings of this study are available within the article and its supplementary materials.
