# Supplementary material for: The usefulness of a checklist approach-based confirmation scheme in identifying unreliable COVID-19-related health information: a case study in Japan
Source: Humanit Soc Sci Commun. 2022 Aug 15;9(1):270. doi: 10.1057/s41599-022-01293-3 (PMC9376898; doi:10.1057/s41599-022-01293-3)
Supplement: Supplementary file 1 — Supplementary Information [file 41599_2022_1293_MOESM1_ESM.docx]

The usefulness of a checklist approach-based confirmation scheme in identifying unreliable COVID-19-related health information: A case study in Japan

**Author information**

**Nanae Tanemura^1^ and Tsuyoshi Chiba^1^**

**^1^National Institutes of Biomedical Innovation, Health and Nutrition, Japan**

**Corresponding author**

**Correspondence to Nanae Tanemura**

E-mail: n-tanemura@nibiohn.go.jp

Questionnaire

1. Main survey

| Following is an article on health information describing the health effects of green tea.  See (2) appendix (Health information) |
| --- |

- (A) After reading this article, do you think tea is effective in preventing infection from the novel coronavirus?

　　 Yes・No

| Following is a flowchart for identifying various types of health information.  See (2) appendix (Explanation)  See (2) appendix (Health information) |
| --- |

- (B) Which step does this article depict?

　　　　Step number referred to from the flowchart here（　　　）

| Following is the answer to the previous question. The correct answer was step (1).  See (2) appendix (Explanation) |
| --- |

| Following is the article on health information regarding the health effects of green tea.  See (2) appendix (Health information) |
| --- |

- (C) After reading this article, do you think tea is effective in preventing infection from the novel coronavirus?

　　 Yes・No

| This is the end of the survey.  Currently, there is no existing evidence that confirms the preventive effect of foods or ingredients on the novel coronavirus infection.  Please note that the health information used in this questionnaire survey has been created for this survey only. |
| --- |

- (D) Participant demographics

| Education level | - Junior high or high school graduate - Junior college graduate - College graduate or higher |
| --- | --- |
| Where do you mostly get health information from?  (multiple answers allowed) | - TV - Radio - Newspaper, magazine, advertisement - Internet - Social networking service - Store front - Hospital - Pharmacy - Drugstore - Contact the company - Family - Friends/acquaintances - Other |
| Frequency of green tea intake | - Every day - Sometimes - Never |
| How would you rate the health benefits of green tea intake? | - Very high - Fairly high - Moderately high - Moderately low - Fairly low - Very low - None |

How easy is it to understand the numerical information shown below?

| Numerical information | Very difficult | Difficult | Somewhat difficult | Easy | Very easy |
| --- | --- | --- | --- | --- | --- |
| Numerical value indicated by “ratio”  (Example: The ratio of women to men is 2:9,998.) | 〇 | 〇 | 〇 | 〇 | 〇 |
| Number indicated by “percentage”  (Example: The salt water concentration is 0.02%) | 〇 | 〇 | 〇 | 〇 | 〇 |
| Precipitation probability of weather forecast  (Example: 20% chance of precipitation) | 〇 | 〇 | 〇 | 〇 | 〇 |
| Calculation of consumption tax  (Example: If the consumption tax is 8%, the sweets at 200 yen are calculated at 216 yen, including tax) | 〇 | 〇 | 〇 | 〇 | 〇 |

1. Appendix

(Health information)

The novel coronavirus disappears upon consuming green tea

**
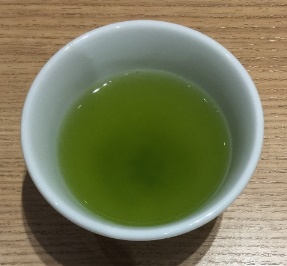
**Every day, the TV and newspapers carry several reports regarding the “medical collapse” crisis. Many researchers have published academic papers on the novel coronavirus. Furthermore, recent studies have also reported surprising research findings. In this article, I will elucidate a study employing green tea to eliminate the novel coronavirus.

Green tea has demonstrated medicinal benefits for all diseases since ages. Professor Shunsuke Chiba of the prestigious Imperial Medical University led a research team that made the sensational discovery that green tea eliminates the infection of the novel coronavirus.

　Professor Chiba and his colleagues performed an experiment where the novel coronavirus was mixed with 10 commercially available green and black tea brands. The amount of infectious virus remaining in the mixture was examined after the mixture was left to stand for 1, 5, and 10 minutes. A surprising change was observed only five minutes after the mixture was created: the infectious viruses decreased by 99.99%. Thus, the virus that came in contact with the green tea was alive but no longer capable of invading and infecting cells.

　This noteworthy research finding was presented at an academic conference in Tokyo. Professor Chiba said, “It was found that the catechin contained in tea leaves eliminates the infectivity of the novel coronavirus.” However, this finding has not yet been published in a paper. “If you drink green tea,” he continued, “the spread of infection in Japan may be suppressed.” Currently, Professor Chiba and his colleagues are performing large-scale clinical trials in Japan with various age groups, and it is expected that reports of these clinical trials will be published in the future.

　At the same conference, Professor Kim from Japan Food University announced the following regarding the effect of green tea extract and catechins on the novel coronavirus: “It is not yet published, but there is no doubt that there is some inhibitory effect.”

　Unfortunately, there is no specific treatment for the novel coronavirus. Currently, existing drugs used to treat other diseases are being diverted. If tea can effectively eliminate the novel coronavirus, it can easily and safely be integrated with daily diet as a preventive measure against the novel coronavirus. The Sangyo Co. Ltd. group, which manages nearly half of the country’s domestic tea, is jointly conducting a research study with Professor Chiba. Therefore, further developments are expected in future research.

Japanese B

April 26, 2021.

(Explanation with a five step-flowchart to identify reliable health information)

　In the second step, the key point about distinguishing the reliability of health information is to verify the type of report that is published in the academic journal.

There are two mediums for academic research presentation: (1) academic conferences and (2) academic journals. At academic conferences, everyone has the right to make a presentation. However, this medium lacks scientific basis, since there is no evaluation system for experts to assess the content of the research results.

On the other hand, experts review the entire research content before publishing its results in academic journals. Later, they determine the value and suitability of the research findings for publishing.

　Based on these five steps, let us focus on the following description in the articles presented in the survey.





■ Professor Chiba

Professor Chiba and his colleagues reported the results of an experiment where the novel coronavirus was mixed with 10 commercially available green and black tea brands.

However, the description below says “…research finding was presented at an academic conference in Tokyo.”

In conclusion, this article was based on a specific research. However, Professor Chiba’s findings have not been published in any academic journals, making the evidence scientifically insufficient.

Let us stop and calmly reflect on this.
